# Protocol for a cluster randomised trial of a communication skills intervention for physicians to facilitate survivorship transition in patients with lymphoma

**DOI:** 10.1136/bmjopen-2016-011581

**Published:** 2016-06-28

**Authors:** Patricia A Parker, Smita C Banerjee, Matthew J Matasar, Carma L Bylund, Kara Franco, Yuelin Li, Tomer T Levin, Paul B Jacobsen, Alan B Astrow, Howard Leventhal, Steven Horwitz, David W Kissane

**Affiliations:** 1 Department of Psychiatry & Behavioral Sciences, Memorial Sloan Kettering Cancer Center, New York, New York, USA; 2 Department of Medicine, Weill Cornell Medical College, New York, New York, USA; 3 Department of Medicine, Memorial Sloan Kettering Cancer Center, New York, New York, USA; 4 Department of Communication Studies, Hamad Medical Corporation, Doha, Qatar; 5 Department of Psychiatry, Weill Cornell Medical College, New York, New York, USA; 6 Divison of Population Science, Moffitt Cancer Center, Tampa, Florida, USA; 7 Department of Medicine, Mamonides Cancer Center, New York, New York, USA; 8 Department of Psychology, Rutgers University, New Brunswick, New Jersey, USA; 9 Department of Psychiatry, Monash University, Clayton, Victoria, Australia

**Keywords:** communication, survivorship

## Abstract

**Introduction:**

Survivors of cancer often describe a sense of abandonment post-treatment, with heightened worry, uncertainty, fear of recurrence and limited understanding of what lies ahead. This study examines the efficacy of a communication skills training (CST) intervention to help physicians address survivorship issues and introduce a new consultation focused on the use of a survivorship care plan for patients with Hodgkin's lymphoma and diffuse large B-cell lymphoma.

**Methods and analysis:**

Specifically, this randomised, 4-site trial will test the efficacy of a survivorship planning consultation (physicians receive CST and apply these skills in a new survivorship-focused office visit using a survivorship plan) with patients who have achieved complete remission after completion of first-line therapy versus a control arm in which physicians are trained to subsequently provide a time-controlled, manualised wellness rehabilitation consultation focused only on discussion of healthy nutrition and exercise as rehabilitation postchemotherapy. The primary outcome for physicians will be uptake and usage of communication skills and maintenance of these skills over time. The primary outcome for patients is changes in knowledge about lymphoma and adherence to physicians’ recommendations (eg, pneumococcus and influenza vaccinations); secondary outcomes will include perceptions of the doctor–patient relationship, decreased levels of cancer worry and depression, quality of life changes, satisfaction with care and usage of healthcare. This study will also examine the moderators and mediators of change within our theoretical model derived from Leventhal's Common-Sense Model of health beliefs.

**Ethics and dissemination:**

This study was approved by the Institutional Review Boards at Memorial Sloan Kettering Cancer Centers and all other participating sites. This work is funded by the National Cancer Institute (R01 CA 151899 awarded to DWK and SH as coprincipal investigators). The content is solely the responsibility of the authors and does not necessarily represent the official views of the National Cancer Institute (NCI) or the National Institutes of Health (NIH). The study findings will be disseminated to the research and medical communities through publication in peer-reviewed journals and through presentations at local, national and international conferences.

**Trial registration number:**

NCT01483664.

## Introduction

There are more than 14.5 million cancer survivors in the USA, this number having tripled over the past 30 years, in part due to advancements in treatment and detection.^1^ On the whole, patients diagnosed with cancer have an estimated 5-year survival rate of 67%.^1^ The definition of a cancer survivor varies, with some applying the term from diagnosis to the end of life as a form of motivation and empowerment.^2^ Many cancer centres use the term survivor for those who have completed primary treatment with curative intent, and we adopt this definition for this project.^3^ Survivors experience multiple challenges, many for which they are unprepared. The Institute of Medicine's (IOM) 2005 report on cancer survivorship, ‘Lost in Transition’, describes this common experience after primary treatment has been completed. Along with late and long-term effects of treatment, survivors are also at risk of recurrence, new cancers and difficulty coping.^2^

Approximately 90% of patients with Hodgkin's lymphoma (HL) are aged 16–65 when diagnosed.^4^ Most patients with limited-stage disease can be cured, and many, even with advanced disease, will also be cured.^4^ Diffuse large B-cell lymphoma (DLBCL) comprises ∼30% of all new diagnoses of non-HL in the USA and other Western nations. The median age of onset is the mid-60s, but among young adults, it is a disproportionately common cancer diagnosis.^5^ With the now standard initial chemoimmunotherapies, the majority (>70%) of patients will be cured.^6^ Complete response rates range from 69% to 85%, with relatively low relapse rates after attaining remission. New and more aggressive regimens may achieve even higher cure rates among high-risk patients.^7^ This excellent prognosis after similar types of treatment makes those completing therapy for DLBCL and HL a relatively homogeneous group of easily identified survivors for prospective studies.

The transition period just after completion of therapy carries heightened distress and many unmet needs for cancer survivors.^8^ ^9^ Lingering physical effects of treatment (eg, fatigue), greater time to reflect on fear of recurrence and decreased contact and support from the health team all contribute. Adult lymphoma survivors have many unmet needs including sexual concerns, medical and living expenses, emotional difficulties, employment problems, and family problems.^9^ Sick leave and disability issues are also common up to 15 years following diagnosis of HL.^10^ Other survivorship concerns reported among individuals with lymphoma include anxiety and concern about remission status.^11^ Moderate distress is common after treatment; severe depression or anxiety occurs in a minority of patients.^12^ ^13^ Lymphoma survivors may also be at risk for late effects of therapy including second primary malignancies, cardiac and pulmonary disease and endocrine dysfunction.^14^

Thus, survivorship care planning appears to be a promising tool to help survivors manage their needs and ensure that they do not fall through the cracks of the healthcare system.^2^ The IOM recommends creation of a follow-up care plan for all cancer survivors, which includes a written summary of the treatment received and a health promotion and schedule for monitoring possible recurrence and other treatment-related risks (ie, survivorship care plan).^15^ The Commission on Cancer Program Standards requires that patients who have completed treatment receive a treatment summary and follow-up plan.^16^ Follow-up programmes for cancer survivors require specialised healthcare by providers familiar with long-term risks, appropriate screening and surveillance, and knowledge about the impact of adoption of healthy behaviours and risk reduction. Sharing a care plan with patients’ primary care physicians can create a team structure with the patient playing a central role, empowered to communicate with practitioners and to continue the process of initiating and integrating healthy behaviours and self-care into daily life.^17^ ^18^ The outcome would be to decrease morbidity from cancer treatments and improved quality of life (QoL),^19^ in contrast to the distress and poor QoL documented among patients who feel that decision-making is out of their control.^20^ Communication skills training (CST) can allow the oncologist to play a critical role in sharing the creation and definition of goals for the post-treatment team.

### Theoretical model

Representations of illness, treatment and post-treatment evaluations of treatment efficacy are generated by the interaction of symptoms, dysfunction and diagnostic statements with existent prototypes or memory models of each, that is, illness, treatment and the plans for management. Each of these ‘common-sense’ representations are abstract, represented by words, and concrete or experiential factors, that is, cancer is a lump, treatment makes one nauseous, and outcomes are evaluated both concretely, for example, did the tumour disappear?, and by input from practitioners, for example, s/he said there is no sign of the cancer. Representations are updated by new information at both the experiential, for example, changes in symptoms and function, and abstract levels, for example, statements from physicians and nurses.^21–23^

Leventhal's Common-Sense Model (CSM) of self-regulation provides a comprehensive explanatory model of illness and treatment that can be shared by patient, physician and family members, allowing reciprocal cross-checking and updating that can create mutual, that is, shared representation of illness, treatment and self as ill and healthy.^22^ Sharing, however, is difficult due to differences in the knowledge base of the participants, for example, the patients symptom experience cannot be directly felt by the physician, and the patient lacks the knowledge base of the physician, biomedical education and contact with multiple patients with similar diseases. Thus, the prototypes or memory structures underlying the current representation of cancer, treatment and the self differ in detail and structure and resistance to assimilating new information.

At entry into cancer survivorship, the physician confronts the need to explore and alter the patient's prototypes of cancer, a transition that can be difficult to achieve as the illness history of many if not most patients is based on experience with acute conditions. Thus, the patient's experience and hoped for outcomes are for cure, that is, the disease is gone, and a return to the precancer self, I am cured and requires a shift into a cancer survivorship prototype. This new understanding moves beyond a focus on follow-up tests to a new concept of survivorship that also includes health promotion and prevention of the long-term and late treatment side effects. The introduction of screening and self-monitoring behaviours alongside radiological and blood tests for early detection of change comprises the preventative and risk-reducing dimension to the postcancer treatment plan of medical care. This shift to a new prototype of illness representation within Leventhal's CSM is aided by the designation of a new visit to serve this agenda, declaring an educational process and describing the key domains of this survivorship prototype: (1) the identity of survivorship as a phase with risk of long-term and late treatment effects; (2) temporal timeline over which these threats can emerge; (3) consequences of delays in recognition or missed opportunities for prevention and risk reduction; (4) causes through which anticancer treatments like chemotherapy and radiation can contribute to secondary cancers and other illnesses and (5) controllability, through which harm can be prevented or minimised via screening and health promotion. The new consultation educates about these, while the ‘summary and care plan for survivorship’ lays out a concrete action plan that the patient is invited to understand, so that their knowledge empowers their perception of control through recognition of risks and pathway to health promotion.

## Methods and analysis

### Study design

Participating sites will be randomised to either the survivorship planning consultation or wellness rehabilitation consultation arms in a multiple-level, cluster-randomised design, which protects against physician contamination of the intervention within any site. We aim to enrol 36 physicians (18 to each study arm).

### Participants and recruitment

*Physicians*: Medical oncologists from each site will be recruited from their respective lymphoma services. After a letter of introduction from the principal investigator (PI), site PIs and each service chief, the investigators will discuss with each physician the project, its documented benefits and time commitments and ask each to sign an informed consent approved by their institution's Institutional Review Board (IRB).

*Patients*: Patients will be recruited through the physicians’ clinics at each of the four sites. Patients are eligible if they: (1) have a diagnosis of HL or DLBCL, as per pathology report or physician assessment in medical record and/or clinical judgement of the treating physician, treated with curative intent; (2) have end of treatment testing indicating, in the opinion of the treating physician, complete remission following completion of chemotherapy and immunotherapy and/or radiation therapy; (3) are at least 18 years old; (4) fluent in English as judged by the consenting professional and (5) able to understand all aspects of the study, provide informed consent and complete all study measures. Patients will be ineligible if they: (1) show evidence of cognitive impairment severe enough to preclude giving permission to the study staff, or completing the survey instruments of the study or (2) have a prognosis and/or comorbidities that, in the physician's judgement, makes them inappropriate for participation.

*Baseline cross-sectional study*: To establish physicians’ baseline communication behaviours, two treatment completion consultations (up to 3 years post-treatment) will be recorded per physician prior to the physician's entering into the assigned intervention. We have chosen this time frame to obtain a pretraining understanding of the physicians’ communication skills when talking with patients with lymphoma during the survivorship period. Approximately 72 patients will be approached in clinics by a member of the research staff from each site. If the patient agreed to participate, we will obtain informed consent and then set up the audio-recorder.

*Longitudinal study*: Once the physician completes training for the survivorship planning consultation or the wellness rehabilitation consultation, the second cohort of patients (N=288) will be recruited and followed longitudinally. With the physician's permission, eligible patients will be contacted during treatment via phone, a mailed letter or in person during a clinic visit. During this initial patient contact, a member of the research staff will discuss the purpose of the study and study procedures with the patient and assess interest. If interested, a member of the research staff will arrange to meet with the patient in clinic prior to their first post-treatment consultation with the physician. At that time, if the patient's physician agrees that the patient meets eligibility criteria, the research staff member will obtain the patient's informed consent to participate. Patients will be asked to stay after this clinic visit to complete questionnaires. If needed, the patient can take the questionnaires home for completion and return via mail. A survivorship planning consultation or wellness rehabilitation consultation appointment will be scheduled within the next month and patients will complete questionnaire assessments after that visit. Patients will complete additional questionnaires after their 3, 6, 9 and 12 month follow-up visits with their oncologists.

### Survivorship planning consultation

Physicians in the survivorship planning consultation arm will complete a 5-hour CST training which consists of: (1) a CST teaching module on empathy that introduces the strategies, skills and tasks that we target in CST through a didactic and exemplary video and (2) the module on survivorship which includes (A) a didactic about survivorship, reviewing the evidence, covering themes for survivors in general and lymphoma survivors in particular, (B) an exemplary video, (C) role-play work about transitioning patients to lymphoma survivorship and introducing the patient to the survivorship care plan and (D) a concluding, reflective discussion about the benefits and barriers to implementation through creating a dedicated consultation focusing on survivorship.

In the second component of the intervention, patients and physicians will participate in a new consultation focused on transitioning the patients to survivorship. In this visit, physicians will review the survivorship care plan and facilitate a discussion about the patients’ concerns related to survivorship. The care plan consists of a written summary of the cancer diagnosis, key test results, staging and prognosis, treatments and relevant toxicities, if they occur, frequency of future visits and surveillance schedule, and review of health promotion behaviours (exercise, nutrition, smoking cessation). Thus, there is coverage of nutrition and exercise to the extent deemed appropriate for each patient, but not to the extensive degree sought in the wellness rehabilitation consultation.

### Wellness rehabilitation consultation

Physicians in the wellness rehabilitation consultation arm will receive a 2-hour training that is focused on wellness and lifestyle factors, with handouts on healthy nutrition and exercise. Physicians in the wellness rehabilitation consultation arm will also have a time-matched 15 min clinical and educational consultation with their patients 1 month after end of treatment is achieved.

Key content includes: (1) review the results of end-of-treatment testing showing that the patient has achieved complete remission, including explanation of residual masses or adenopathy, and congratulate the patient for having achieved a complete remission, (2) conduct any appropriate physical examination, (3) discuss the benefit of healthy nutrition and give the handout sheet as a guide, (4) discuss a graduated walking programme to promote fitness and provide an exercise sheet as a guide, (5) invite questions, (6) review any medications, (7) invite the patient to get in touch with any concerns and (8) plan a 3-month follow-up appointment. The physician is free to answer all questions fully and appropriately. Audio-recording will enable the study researchers to code for the content of discussions.

*Consolidation of training in the survivorship planning or wellness rehabilitation consultations*: In both study arms, when a physician's consultation coding reveals that <80% of defined behaviours are achieved, a study investigator (TTL/MJM/SH) will contact the physician via telephone, in-person meeting and/or email. This discussion will focus on both learner-nominated issues plus feedback on coded consultations to reinforce the use of strategies, tasks and skills, discussion of barriers and situation-specific suggestions for skills uptake. Such supervision is part of achieving fidelity of the intervention. Fidelity coding of intervention sessions will occur throughout the study.

### Assessment and evaluation plan

*Pretraining use of communication skills*: After randomisation, to assess the baseline approach used by physicians during survivorship visits, two consultations with patients who have completed treatment for lymphoma (up to 3 years post-treatment) will be audio-recorded and coded for physicians in both arms of the study. The goal of this cross-sectional study will be to describe the content and nature of current communication behaviours during follow-up visits of patients with HL and DLBCL within the patients’ first 3 years of survivorship. Physician characteristics including age, gender and years of experience will be collected at baseline. This cross-sectional study will be written up descriptively.

### Assessment of physicians

Survivorship planning consultation arm physicians will participate in a pretraining and post-training Standardized Patient Assessment (SPA) to demonstrate uptake of skills in the discussion of survivorship. After training in the survivorship planning consultation or wellness rehabilitation consultation, physicians in both arms will be audio-recorded three times with visits from newly enrolled patients with lymphoma, first at the initial postregistration consult, then for the survivorship planning consultation or the wellness rehabilitation consultation, and finally at the 3-month follow-up visit so that the maintenance of skills can be assessed.

### Standardized Patient Assessments

Physicians in the survivorship planning consultation arm complete SPAs that are video-recorded before and after they participate in the CST training. Blinded coding will be completed by double-blind coders using the Comskil Coding System (CCS) and the Specific Coding Schema for Skills, Strategies and Process Tasks developed for the Transition to Survivorship module. The SPA is a reliable assessment with discriminant validity.^24^

#### Clinical consultation assessment

After training, for the physicians in the survivorship planning consultation arm, ∼8 consecutive, consenting patients with lymphoma per physician in which the transition to survivorship is discussed will be audio-recorded in the naturalistic setting: first at the initial postregistration visit when treatment completion is identified, then 1 month later at the survivorship planning consultation, and finally at the 3-month follow-up visit. For the wellness rehabilitation consultation physicians, ∼8 consecutive, consenting patients with lymphoma per physician will be recorded at the initial post-treatment visit, 1 month later as an attention-time follow-up (nutrition and exercise) and again at a 3-month follow-up.

*The Comskil Coding System*: The CCS is a schema of the strategies, skills and process tasks taught explicitly in our modules and used extensively in our standard curriculum. The CCS codes verbal utterances (skills) when present (such as declare agenda, check understanding, encourage expression of feelings, etc), but does not code non-verbal behaviours.^25^ Inter-rater reliability for CCS (measured using Cohen's κ) has been between r=0.76 and r=0.84.^24^ An additional coding manual covering seven task categories was developed and piloted for the survivorship planning consultation module. The seven task categories include (1) use of a survivorship care plan, (2) disease and treatment details, (3) long-term effects discussion, (4) potential late effects discussion, (5) specific physician recommendations, (6) additional health maintenance recommendations and (7) possible social issues discussion.

*Fidelity of CST intervention facilitation*: To ensure uniformity of CST training across the two arms (survivorship planning consultation and wellness rehabilitation consultation), each CST facilitator will have their role play sessions audio-recorded and coded for fidelity to the model using the Comskil Facilitator Assessment Coding System (C-FACS). Inter-rater reliability has been demonstrated at κ=0.82 and our facilitators maintain >80% adherence.^26^ Feedback will sustain attention to the model, as well as facilitators’ briefing and debriefing sessions pretraining and post-training.

*CST course evaluations*: After each training workshop, physicians will fill out an evaluation rating their confidence in using the behaviours taught. Although administered post-training, these items ask physicians to rate their confidence in dealing with these issues before and then as a result of training.

### Assessment of patients

We have selected reliable and well-validated measures of patient knowledge, worry about recurrence, depression, QoL, sexual functioning, perception of their physician's empathy and satisfaction with the consultation to enable us both to explore key patient outcomes and examine the moderators and mediators of change over time. Demographic data and contact information will be obtained at baseline from consented patients. Medical and treatment information will be extracted from the electronic medical record during the study. Adherence outcome data will be obtained from the Employment and Health Services Questionnaire (EHSQ). Patient questionnaires are completed immediately after their first post-treatment visit, after their survivorship planning or wellness rehabilitation consultation visit (1 month later) and then immediately after their 3-month, 6-month, 9-month and 12-month follow-up visits.

*Patient medical assessment*: The medical and treatment data will be extracted from the patient record.

*Patient demographic form*: Patients will be asked to indicate their current work status, marital status, religion, education level, race, ethnicity and country of birth.

*Lymphoma Knowledge Questionnaire*: This 50-item questionnaire examines understanding of causes, treatments, late effects and care needs for patients with HL and DLBCL, and items were distributed equally across five levels of difficulty. After iterations to modify to a suitable level of health literacy, it was administered to 320 respondents, confirming its face validity and ease of comprehension.

*Cancer Worry Inventory*:^27^ The Cancer Worry Inventory (CWI) is a 24-item scale assessing worries across the following domains: health or physical illness, work, financial, religious or spiritual, family or friends, social and leisure activities, sexuality, self-appraisal and existential concerns. Internal consistency by Cronbach's α was 0.93, with five factors ranging from 0.76 to 0.92.

*Patient Health Questionnaire-9*:^28^ This nine-item well-validated measure uses the items that form a Diagnostic and Statistical Manual of Mental Disorders, Fourth Edition (DSM-IV) diagnosis of depression.

*Quality of Life Cancer Survivor*:^29^ The Quality of Life Cancer Survivor (QOL-CS) is a 41-item instrument that assesses four QoL domains: physical, psychosocial, social and spiritual well-being. Test–retest reliability is high, r=0.81–0.90 and Cronbach's α is 0.93. Its total score correlates with the Functional Assessment of Cancer Therapy-General (FACT-G) at r=0.74.

*Sexual functioning*: Males—the SEAR^30^ is a 14-item, five-point Likert response scale that assesses sexual satisfaction, sexual self-esteem and overall relationship satisfaction. Cronbach's α values cover 0.76–0.93 and validity includes good sensitivity to change in men treated for sexual dysfunction. Females—the Female Sexual Functioning Index (FSFI)^31^ is a 19-item scale that assesses female sexual function in five domains: (1) desire and subjective arousal, (2) lubrication, (3) orgasm, (4) satisfaction and (5) pain/discomfort. The test–retest reliability of the FSFI is 0.88 and the internal consistency is 0.89–0.97. Discriminant validity is significant across a wide range of ages and discerns sexual dysfunction readily.

Consultation and Relational Empathy (CARE)^32^ is a well-validated 10-item self-report measure of a patient's perspective of physician empathy. It focuses on emotional, cognitive and behavioural aspects crucial to patient-centredness. In 710 patients with cancer, empathy was an important pre-requisite for information provision, with key effects on development of depression and social–emotional–cognitive QoL. Busyness had the strongest negative influence on physician empathy. An additional eight items have been added for follow-up consultations that will examine aspects of Leventhal's CSM of illness, revealing the patient's view about how helpful the physician was at providing help with fears of recurrence, future expectations, tips for getting on with life, future anticancer screening plans, self-monitoring, high-risk behaviours, exercise and nutrition.

Patient Satisfaction with the Consultation (PSC) is a well-validated 25-item, five-point measure^33^ ^34^ to assess satisfaction with: (1) amount and quality of information; (2) emotional support and (3) patient participation. Cronbach's α is 0.91. The PSC has demonstrated sensitivity to behavioural changes like meeting involvement preferences.^35^

*Physical Activity^36^,^37^ and Nutrition^38^ Measures*: The Physical Activity and Nutrition Measures (PANM) assesses the frequency and average duration of mild, moderate and strenuous levels of physical activity. Similarly, the frequency and quantity of vegetable and fruit intake are reported to measure dietary intake.

*Cancer Behavior Inventory, Brief version*:^39^ This 14-item measure assesses self-efficacy in four areas: maintaining independence and positive attitude; participating in medical care; coping with stress; and managing affect related to cancer. The brief version was validated in 735 participants with cancer in the USA.

*Employment and Health Services Questionnaire*: This 26-item survey assesses work status (lost workdays, difficulty concentrating); usage of health services; impairment in performing activities (eg, school, taking care of family, volunteer work); use of emergency and urgent care services; hospitalisations; visits, phone calls and email contacts with the oncologist or cancer treatment team; visits with other doctors; X-rays; or scans such as CT and MRI; adherence to recommended vaccinations and anticancer screening tests; and visits with psychologists, social workers or other counsellors.

*Usage of summary care plan*: This 3-item measure assesses usage of the summary care plan by the patients in the survivorship planning consultation arm of the longitudinal phase of the study. The questions ask about personal use of the care plan after the initial consultation with the physician, and sharing of the document with friends, relatives and other clinicians. This questionnaire will only be completed by patients in the survivorship planning consultation arm of the study, as patients in the other arm of the study do not receive a care plan.

*Qualitative interview schedule to examine development of the CSM of cancer survivorship*: This 22-item questionnaire has been developed with Dr Leventhal to explore elements of the CSM survivorship prototype, including employment history and current status, participation in activities and chores around the house, barriers in sustaining pretreatment level of activity, actions taken to achieve pretreatment level of activity, participation in leisure activities, worry of cancer recurrence and preventive behaviour adopted, and things/activities patients are initiating to optimise wellness. The interviews will be analysed both qualitatively and quantitatively to provide an understanding of the explanatory model, as well as to also highlight coping behaviours and preventive actions adopted by patients to sustain health.

Each site will enter patient information into a secure database. Questionnaires will be scanned and sent via secure email to the main site. All data will be stored in locked cabinets. There will be regular conference calls for the study investigators at all sites to discuss project details and any protocol modifications.

## Statistical plan

The overall analytic strategy for this cluster-randomised clinical trial^40^ will be based on a linear mixed-effects modelling approach (cite Laird and Ware; also known in behavioural sciences as the Hierarchical Linear Modeling approach^41^ because of the hierarchical nature of the data. Postintervention assessment(s) are nested within individual patients, patients nested within clinicians and clinicians nested within participating sites. It is a *hierarchical* data structure because physicians acquire communications skills, and the effects of the acquired skills would cascade down to benefit patient outcomes. There are two general types of outcomes: (1) outcomes at the level of clinician trainees; and (2) outcomes at the level of individual patients. The nested hierarchical data structure introduces intraclass correlations (ICCs) within clusters such that, for example, patients who see the same physician are likely to show correlated outcomes and clinicians working at the same hospital sites may also show correlated skill uptakes. Mixed-effects modelling takes into consideration the ICCs due to the nesting. The assumption of independent observations, such as that required by independent-sample t-test and analyses of variance, is not tenable.

There are two types of outcomes in the hierarchical data structure—outcomes at the level of physicians and outcomes at the level of individual patients. The primary outcome for physicians is uptake and usage of communication skills, determined as the composite scores of the cumulative use of communication skills coded from the three recordings of actual patient consultations post end of treatment, and maintenance of these skills at 3 months postintervention. For each physician, we will have recordings of ∼8 patients after the survivorship planning consultation or the wellness rehabilitation consultation.

The primary outcome for patients, assessed at the 12 months time point, is change in knowledge about lymphoma (a continuous variable) and adherence to physicians’ recommendations (dichotomous outcomes). The secondary patient outcomes include cancer worry, QoL changes, satisfaction with care and usage of healthcare. This study will also examine moderators and mediators of change within our theoretical model derived from Leventhal's CSM of health beliefs. Each patient's adherence outcome will be a percentage of accomplished over recommended behaviours at the final assessment point, where the number of recommendations will have been tailored to each individual's needs.

The specific analytic strategies to address the research study aims are outlined as follows:

*Aim 1* : To determine the impact on the physicians’ communication skills uptake on transitioning patients with lymphoma from treatment to survivorship.

A linear mixed-effects model will be used to address this aim at the level of enrolled physicians. The effective sample size testing the superiority of communications skills will be the number of enrolled physicians, clustered into physicians who were randomised into the survivorship care planning arm and physicians randomised to the wellness rehabilitation arm. This hypothesis will be tested by a fixed treatment effect, taking into consideration random effects of sites and the physician within the sites. For the maintenance of skills, a similar mixed-effects model will be used to estimate the extent to which survivorship planning consultation confers greater skills maintenance than the wellness rehabilitation consultation.

*Aim 2* : To determine the impact on patient outcomes of the survivorship planning consultation intervention.

A slightly more complex, two-level linear mixed-effects model addresses this aim to take into consideration patient-level outcomes nested within physicians, and physicians nested within participating sites. The effect of the survivorship planning consultation on patient outcomes will be evaluated by a fixed effect of training, and effects attributable to hierarchical data will generally be modelled as random effects (eg, individual patients and the sites).

At the patient level, we may include the repeated assessments of patient outcomes, a maximum of six repeated outcomes if there are no missing data and, depending on the amount of available data per patient, a growth-curve analysis may be possible, although this analytic approach has to be evaluated empirically, depending on the observed pattern of data attrition.

To better account for individual differences in skills, change scores may be calculated (ie, postintervention score minus baseline score of the same domain) and entered into the statistical model as the outcomes of interest. Change scores have the advantage of creating easily interpretable results and clearly indicating the direction of individual change. The primary hypothesis is supported if the difference between the two study arms is statistically significant. Additional baseline covariates may be considered for inclusion at the patient level (eg, age, sex, ethnicity and disease stage at time of diagnosis) as well as at the physician level (eg, physician's seniority, standardised patients’ assessments). Inclusions of covariates typically reduce residual errors and boost statistical power by adjusting for physician heterogeneity.

The primary adherence domain will be health promotion behaviours indicated by guidelines for age, gender and other guideline recommendations, including mammography, Pap smear, colonoscopy, prostate-specific antigen, influenza and pneumococcus vaccines. For example, colonoscopy screening may be indicated for patients older than 50 years of age who have not had a colonoscopy within the past 10 years.

*Aim 3* : To explore moderators and mediators of improved patient outcomes. We predict that greater levels of empathy in the consultation and deeper understanding of the survivorship and care plan will mediate reduced patient worry. Moderating effects will be addressed through the inclusion of interactions between the intervention indicator variable and moderators such as age, race, ethnicity and other sociodemographic variables. Mediating effects require a path analysis model or a generalised latent variable modelling approach,^42^ using the statistical packages LISREL^43^ or AMOS.^44^ Choice of appropriate statistical tests of mediating effects will be guided by MacKinnon *et al*^45^ (eg, model equivalence to examine whether or not the mediating path model is equivalent across patients nested within physicians who received different interventions). For each physician, we will calculate a change in empathy score from the CARE questionnaire before and after the intervention or for the same yoked time period. The change in empathy will be used as the mediator. To test hypothesis 3, we will fit a multi-level modelling (MLM) similar to the previous analyses with patient worry as the dependent variable. An interaction between intervention and change in empathy will be tested as well. Hypothesis 3 will be supported if we observe a statistically significant interaction between intervention and change in empathy.

### Statistical power and sample size considerations

At the end of the study, we anticipate to have patient-level data from ∼7 out of the 8 consented patients per physician (80% retention at the patient level) and 32 out of the 42 participating physicians (88% retention at the physician level). Using the formula in Donner and Klar^46^ for cluster-randomised trials, we estimated the statistical power that can be attained by sampling 7 patients from 32 physicians (16 in each arm). We estimated statistical power on knowledge as well as adherence at a two-sided type-I error rate of 0.05, and an overall ICC of 0.25 between members of the same cluster. First, we summarise the statistical power estimates for patients’ knowledge about lymphoma. A meta-analysis of studies examining the effect of education interventions for knowledge^47^ with a combined total of over 5000 patients with cancer, found a large effect size of 0.90 (95% CI 0.61 to 1.20) for knowledge. We have powered the present study conservatively with an effect size estimate of 0.61, the lower bound of the CI. We will have an 80% statistical power to detect an effect size of 0.61. We assumed an ICC of 0.25 among patients nested within physicians, a conservative estimate compared with prior studies which showed typical values of ICC of 0.002–0.012.^40^

Similarly, we will be able to detect an effect size of 0.61 in health screening adherence. We illustrate the anticipated difference in the adherence rates across the two study arms. Patients of physicians in the wellness rehabilitation consultation arm may have an adherence rate of 50% of patients meeting the dichotomised adherence criterion above. An effect size of 0.61 translates to a 78.5% or greater health screening adherence among patients of physicians in the survivorship planning consultation arm by Cohen's formula.^48^

Several statistical details will have to be addressed empirically, after we have fully described the amount of data available for analysis. For example, there is likely to be some variability in the health promotion adherence outcomes at the patient level, due in part to the variability in the appropriateness in individual recommendations (eg, colonoscopy only appropriate if age ≥50). Hence, the analytic strategy will have to take into consideration such unpredictable circumstance. Mixed-effects modelling is highly flexible in accommodating these variabilities.

## Ethics and dissemination

All participants will provide informed consent and may withdraw at any time without impacting their treatment or relationship with their clinical team. Study results will be presented at national and international meetings and through peer-reviewed publications.

If efficacious, this novel survivorship consultation planning intervention has the potential to change clinical practice for how to transition patients into the survivorship phase of their care. This model could subsequently be modified to be implemented with other patient populations with cancer. This new standard of care has the potential to enhance the survivorship experience, well-being and QoL in patients newly free of cancer.
